# An Interesting Case of Critical Spontaneous Ureteral Rupture

**DOI:** 10.7759/cureus.17497

**Published:** 2021-08-27

**Authors:** Saad Bin Jamil, Mian Munir, Iqra Patoli, Sameerah Rehmani

**Affiliations:** 1 Hospitalist Medicine (internal Medicine), Saint Mary's Hospital, Waterbury, USA; 2 Internal Medicine, Saint Mary's Hospital, Waterbury, USA; 3 Internal Medicine, Sentara Obici Hospital, Suffolk, USA

**Keywords:** ureteral infection, percutaneous nephroureteral catheter tube, spontaneous ureteral rupture, urerteral rupture, ureteral calculi, severe sepsis

## Abstract

Spontaneous rupture of the ureter in an uncommon presentation. We present a case of an 85-year-old female patient with a past medical history significant for hypertension and hyperlipidemia who presented to the emergency room (ER) due to abdominal pain and nausea. Computed tomography (CT) of abdomen and pelvis revealed proximal ureteral and ureteropelvic junction rupture with fluid within the left retroperitoneum and pelvis. No clear etiology was identified. The patient had a left percutaneous nephroureteral catheter tube (PNCT) placed in addition to being given broad-spectrum antibiotics for possible infection. She was noted to improve and was discharged with outpatient follow-up for tube removal.

Our patient presented with a rupture of the ureter; however, the source remained elusive. CT scan assisted with the diagnosis but there is no evidence of hydronephrosis or mass. There are no clear treatment guidelines for spontaneous ureteral rupture as the presentation is rare. Treatment may involve percutaneous drainage and possibly antibiotics for concurrent infection. Surgical intervention may be required in cases where severe complications arise. Early diagnosis and management may prevent long-term morbidity and mortality.

## Introduction

Ureteral perforation is a rare condition and can lead to complications such as urosepsis, formation of abscess, retroperitoneal urinoma, and renal dysfunction [1]. There are only a few reported cases of spontaneous ureteral rupture, which is a rare entity [2]. Common causes of spontaneous ureteral rupture include strictures, cancer, or nephrolithiasis. Although spontaneous ureteral rupture may not always be symptomatic, it can lead to severe complications, which can lead to morbidity and mortality. Symptoms may include abdominal tenderness, nausea, or vomiting. 

Diagnostic modalities include excretory or retrograde urography, computed tomography (CT) scan, or magnetic resonance imaging (MRI). Due to the rarity of presentation, there are no clear established treatment guidelines based on randomized clinical trials. Different treatment options can be pursued based on the patient's overall clinical status. In patients who are hemodynamically stable, supportive care may be adequate. However, patients with complications may require more acute intervention such as percutaneous nephrostomy (PN) or open surgical approach. Patients may have a concurrent infection in the setting of ureteral rupture which may warrant treatment with antibiotics.

We present an interesting case of spontaneous ureteral rupture with no clear pathological etiology, which led to significant complications requiring an intensive level of care. The case highlights the importance of timely identification as well as diagnostic modalities and treatment options for patients who present with spontaneous ureteral rupture.

## Case presentation

An 85-year-old woman with a past medical history of hypertension, hyperlipidemia, cerebrovascular accident (CVA), and left lower lung lobectomy presented to the ER due to intermittent abdominal pain, dysuria, and nausea for one day. Physical exam was significant for diffuse tenderness to palpation, worse in the suprapubic region. Her vitals were blood pressure of 167/78 mm Hg, respiratory rate of 16 per minute, pulse rate 87 beats per minute, and temperature of 95.9 °F. Relevant laboratory workup is mentioned in Table 1.

**Table 1:** Relevant Laboratory Results

Laboratory Test	Value	Normal
Leukocyte Count (K/µL)	20.6	4-10.5
Segmented Neutrophils (%)	87	25-62
Urine Analysis: Leukocyte Count	25	Negative
Urine Analysis: White Blood Cell Count (count/hpf)	21-50	0-5
Anion Gap (mEq/L)	15	5-14
Blood Cultures	Escherichia Coli	No Growth

**Figure 1:**
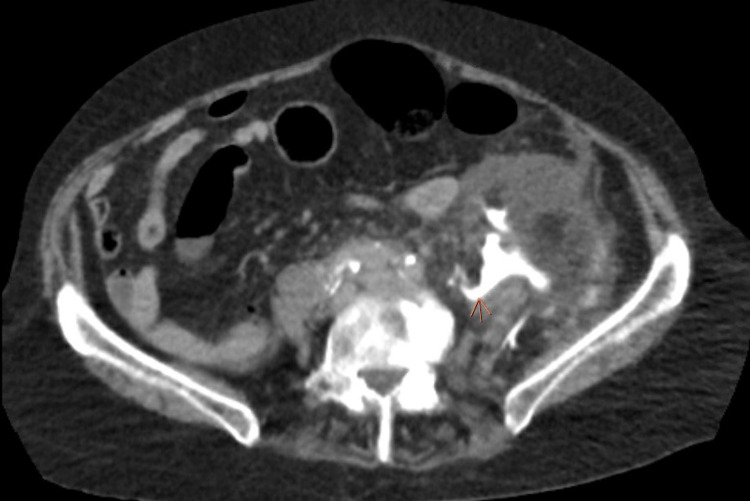
CT abdomen and pelvis showing excreted free contrast seen leaking into pelvis (orange arrow).

CT scan of the abdomen and pelvis with intravenous (IV) contrast was suggestive of proximal ureteral/ureteropelvic junction rupture and a large amount of fluid in the left retroperitoneum and surrounding the left kidney in the pelvis (Figure 1). No clear obstructive etiology was identified on the CT scan. There was no evidence of stone or mass on the CT scan.

The patient received broad-spectrum antibiotics in ER. While the patient was in the ER, she was noted to have an atrial flutter with rapid ventricular rate and systolic blood pressure dropped to 89 mm Hg. She was given an amiodarone bolus and she reverted to normal sinus rhythm. Norepinephrine infusion was also planned to be given but once she reverted to sinus rhythm, her blood pressure was noted to improve. She was also noted to drop her oxygen saturation to 70% and was placed on two liters of oxygen through a nasal cannula.

**Figure 2:**
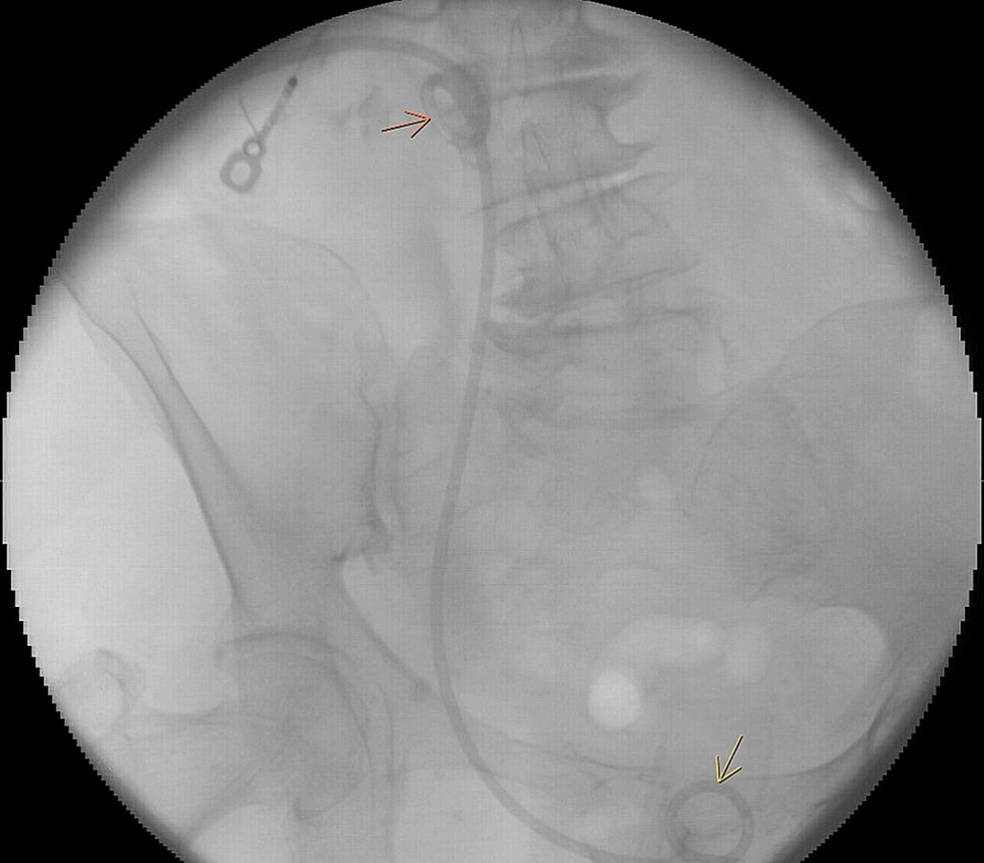
PNCT proximal loop seen in proximal renal pelvis (orange arrow) and PNCT distal loop seen in bladder (yellow arrow).

Urology consultants were consulted and the recommendations were to admit the patient to the intensive care unit (ICU) and placement of percutaneous nephroureteral catheter tube (PNCT). This was done successfully (Figure 2). The patient was monitored in ICU and continued on antibiotics.

**Figure 3:**
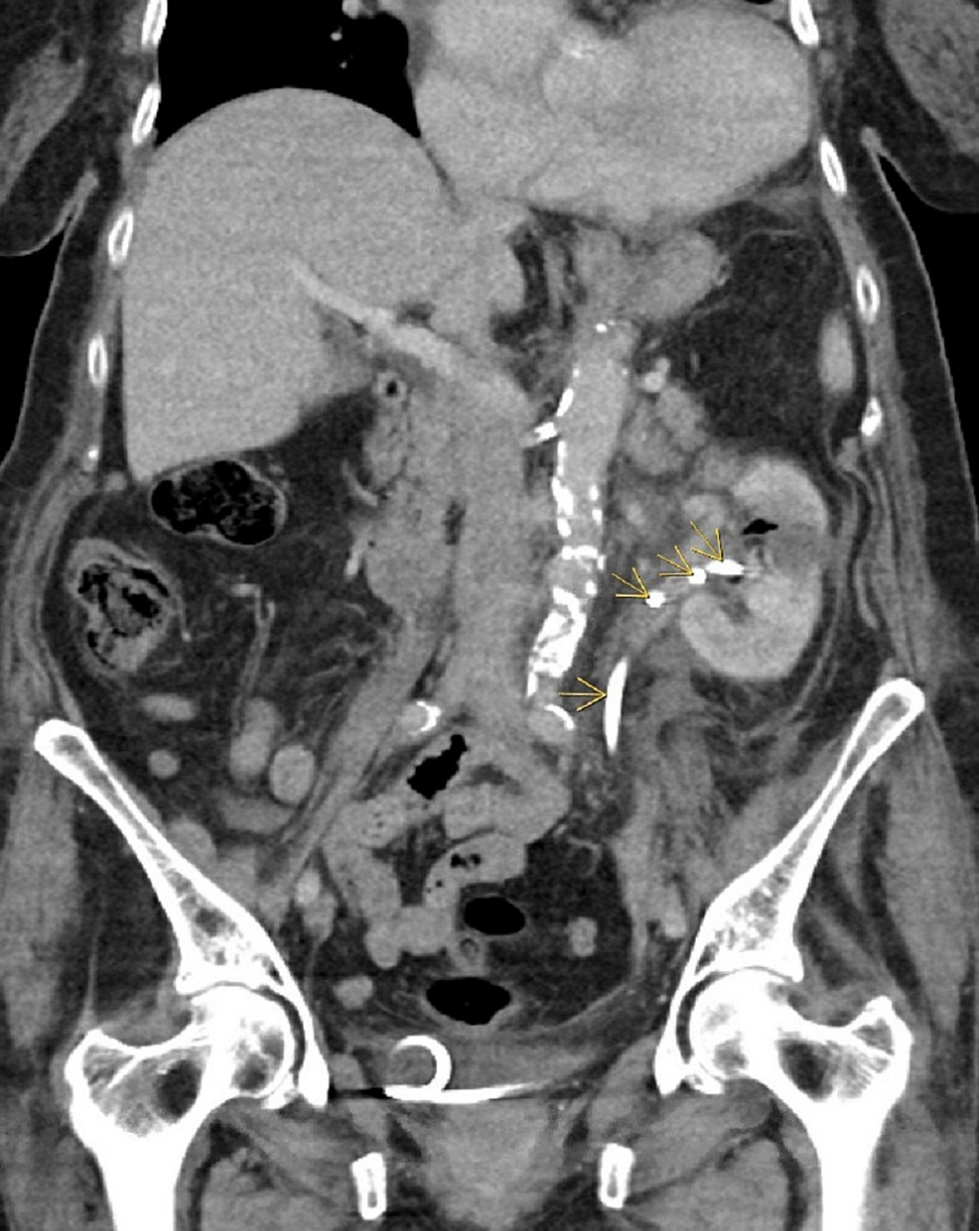
CT abdomen and pelvis coronal view showing PNCT (yellow arrows). PNCT - percutaneous nephroureteral catheter tube

Over the course of her stay, the patient clinically improved, and she was transferred out of ICU to medicine service. Due to persistent leukocytosis, a CT scan abdomen and pelvis with IV contrast was repeated, which did not show any clear infectious etiology (Figure 3). The infectious disease was also evaluated and antibiotics were switched to ceftriaxone as per blood culture sensitivities.

Eventually, the patient's leukocyte count down trended. Clinically she was noted to improve significantly. Urology recommended that PNCT should be kept in place for at least three weeks. She was discharged on oral antibiotics and recommendations were given to follow-up outpatient with urology and IR specialists.

## Discussion

Ureteral rupture can be traumatic or non-traumatic. Spontaneous ureteral rupture is a rare condition with only a few reported cases. Common causes include tumors, strictures, and ureteral calculi [3]. The presentation can vary from abdominal pain, nausea, vomiting, and even sepsis or shock if a concurrent infection is present. 

Cystoscopy and retrograde pyelogram can be urgently performed for diagnosis and stent placement if needed [4]. In cases where these can not be performed, CT with IV pyelogram is the preferred diagnostic modality [4]. “Although rupture can occur at any level of the urinary system, the most commonly described rupture site is the renal fornix; rupture at the level of the ureteropelvic junction (UPJ) is rare” [5]. Regardless of etiology, ureteral rupture should be taken seriously due to potential complications. Some of these complications include urinomas, urosepsis, and formation of abscess [5].

As spontaneous ureteral rupture is a rare condition, there is no clear established guideline or management protocol. Treatment modalities are on a case-by-case basis. Surgical intervention or conservative route can be pursued based on overall clinical status. Factors such as urinary extravasation with urinoma formation, sepsis, and hemodynamic status may determine the potential approach. 

Davies et al. [6] believe that in urinary extravasation, treatment should be supportive initially (i.e., careful observation and analgesics). However, the approach may need to change in the presence of concurrent complications. Acute treatment options include a ureteric stent or PN and open surgical intervention in addition to supportive care. Concurrent infection must be treated as well.

In our patient, there was no clear cause or etiology which was identified despite repeated CT scans. However, ureteral rupture led to complicated urinary tract infection, gram-negative bacteremia, and hemodynamically unstable arrhythmia. This led to the urgent placement of IR-guided left PNCT. More acute intervention may be required if patients have severe pain, concurrent infection, and worsening renal function [7]. “Minimally invasive endourological procedures with double-J catheter placement and percutaneous drainage offer excellent results” [2]. In addition, the patient was given broad-spectrum antibiotics due to sepsis which can occur due to rupture and leakage of infected urine. In our patient, PNCT was pursuing due to ureteral rupture leading to significant retroperitoneal fluid in which case double-J catheter may not be adequate. In addition, there may be a risk of possible worsening of the ureteral tear with the conversion of partial tear into full tear with double-catheter and stent placement. Our patient clinically improved with PNCT and antibiotics. Urology recommended keeping the PNCT in place for at least three weeks and follow-up outpatient to do a nephrostogram to determine if the ureter sealed in which case the PNCT could be removed.

## Conclusions

Spontaneous ureteral rupture is a rare presentation and can occur due to multiple causes. Sometimes, there is an identifiable etiology. ER specialists and urologists should determine management modalities based on a case-by-case basis. Patients with complications should be treated more acutely in addition to the treatment of concurrent sepsis with antibiotics if present. Early diagnosis and management may prevent long-term morbidity and mortality.
